# The Neurobiology of Addiction: An Overview

**Published:** 1997

**Authors:** Amanda J. Roberts, George F. Koob

**Affiliations:** Amanda J. Roberts, Ph.D., is a research associate in the Department of Neuropharmacology, The Scripps Research Institute, La Jolla, California. George F. Koob, Ph.D., is a professor and director of the Division of Psychopharmacology, Department of Neuropharmacology, The Scripps Research Institute, La Jolla, California, and adjunct professor in the Departments of Psychology and Psychiatry, University of California. San Diego, California

**Keywords:** AOD dependence, compulsion, AOD withdrawal syndrome, AOD craving, positive reinforcement, AODD (alcohol and other drug disorders) relapse, AOD abstinence, neurobiological theory, neurotransmitters, neurotransmission, AOD sensitivity, biological adaptation, brain, dopamine, nucleus accumbens, literature review

## Abstract

Addiction can be defined in part as a compulsion to use alcohol or other drugs and the occurrence of withdrawal symptoms when long-term consumption ceases. In addition to physical symptoms related to nervous system hyperexcitability, withdrawal includes changes in mental state that may motivate renewed AOD consumption. The manifestations of addiction are associated with changes in nerve cell function by which the brain attempts to adapt to a drug’s presence. These functional changes modulate a person’s initial response to a drug, the establishment of long-term craving for the drug (i.e., addiction), and the persistent sense of discomfort that leads to relapse after abstinence has been achieved. Research is beginning to reveal how specific brain regions may be integrated to form neural circuits that modulate aspects of addiction.

Addiction can be defined from a behavioral viewpoint as repeated self-administration of alcohol or other drugs (AOD’s) despite knowledge of adverse medical and social consequences and attempts to abstain from AOD use. Typically, an addicted person’s daily activities are centered on obtaining and consuming the drug at the expense of social and occupational commitments. Many factors contribute to the development of addiction. A person’s initial decision to use a drug is influenced by genetic, psychosocial, and environmental factors. Once it has entered the body, however, the drug can promote continued drug-seeking behavior by acting directly on the brain.

Research over the past two decades has increased our understanding of the neural processes that underlie drug-seeking behavior. This article summarizes some of the molecular and cellular events in the brain that appear to be associated with addiction. The article first discusses some observable manifestations of addiction and basic mechanisms involved in initiating and maintaining addictive behavior. The hypothesized roles of various chemical communication systems of the brain (i.e., neurotransmitters and receptors) are explored, followed by a discussion of the interactions between these systems within brain regions thought to be involved in addiction.

Finally, the article discusses the suggested role of an integrated system of neural connections involving several adjacent brain regions. This article is not an exhaustive overview, but a sampling of some topics of interest to researchers studying addiction neurobiology.

## Basic Mechanisms of Addiction

Two characteristics are common to most definitions of AOD addiction: the compulsion to use a drug, leading to its excessive and uncontrolled consumption, and the appearance of a cluster of symptoms when the drug is withheld after a period of its continuous consumption (i.e., withdrawal syndrome). Physiological symptoms of alcohol withdrawal begin from 6 to 48 hours after the last drink and include tremors, elevated blood pressure, increased heart rate, and seizures. AOD withdrawal also includes changes in mental state (e.g., anxiety, negative emotional state, and craving) that may motivate renewed AOD consumption. These signs may both precede and outlast the physiological symptoms. For the purpose of this article, addiction is defined as a loss of control over AOD use and the appearance of a withdrawal syndrome—with motivational aspects—upon cessation of such use.

Two factors that modulate behavior—reinforcement and neuroadaptation—contribute to the addictive process. Reinforcement is a theoretical construct by which a stimulus (e.g., an unconditioned stimulus, such as the drug itself or drug withdrawal, or a conditioned stimulus, such as drug-taking paraphernalia) increases the probability of a response (e.g., continued use of the drug). Neuroadaptation refers largely to the processes by which initial drug effects are either enhanced (i.e., sensitization) or attenuated (i.e., counteradaptation) by repeated AOD exposure. Drug-related responses (i.e., reinforcement) are modulated by the neuroadaptive changes that occur with AOD exposure. Working together, these factors appear to motivate the initial, short-term (i.e., acute) response to a drug and the establishment of the long-term (i.e., chronic) craving for the drug that characterizes addiction. In addition, some neuroadaptive changes may be permanent, producing the persistent sense of discomfort during abstinence that leads to reinstatement of drug use (i.e., relapse).

### Reinforcement

Several sources of reinforcement may contribute to addiction. In positive reinforcement, a rewarding stimulus (e.g., AOD-induced euphoria) directly increases the probability of a response (e.g., continued AOD use). In negative reinforcement, the incentive for AOD use is relief of a painful or unpleasant state (i.e., the physiological and motivational symptoms of withdrawal). In addition to their direct reinforcing effects, drugs can motivate behavior indirectly through environmental stimuli with which the drugs have become associated (i.e., conditioned reinforcement). For example, the locations where drugs are taken or the paraphernalia used for their administration may themselves elicit a druglike state of euphoria in the absence of the drug (i.e., conditioned positive reinforcement). Conversely, exposure to stimuli associated with periods of abstinence may produce symptoms of withdrawal (i.e., conditioned negative reinforcement).

Researchers can examine the reinforcing effects of AOD’s by measuring the behavior of animals exposed to drugs in the laboratory (see figure). A commonly employed method is direct self-administration whereby an animal is either allowed free access to AOD’s (e.g., given a bottle containing alcohol to drink) or required to perform a specific behavior to obtain AOD’s (e.g., trained to press a lever for a small volume of alcohol). Changes in the patterns of self-administration that occur with long-term AOD exposure or following the experimental manipulation of a particular neural system may reveal underlying mechanisms of reinforcement (figure A).

A second behavioral test used to measure the reinforcing effects of AOD’s is intracranial self-stimulation (ICSS). In this procedure, electrodes are implanted in the brain of a rat. The rat is subsequently allowed to press a lever to receive mild electrical pulses through the electrodes (figure B). Animals will self-administer electrical stimulation to certain brain regions at extremely high rates, indicating that such stimulation is reinforcing. Scientists believe that ICSS directly activates the brain’s reward centers, thus providing a unique tool for investigating reinforcement processes. AOD’s appear to make ICSS *more* rewarding by decreasing the amount of current required by the animal to achieve the same level of reward. This ability corresponds closely to a drug’s potential for abuse.

A third behavioral paradigm used to test the reinforcing actions of AOD’s is place conditioning (figure C). In a simple place-conditioning test, an animal is alternately placed in two distinct environments, neither of which initially elicits any apparent behavioral response (i.e., neutral environments). The animal is conditioned to associate one of the environments with the effects of the drug under study. For example, the animal may be placed in a dark chamber with a rough-textured floor after receiving a drug injection, and placed in a light chamber with a smooth-textured floor after receiving an injection of drug-free saline solution. This procedure is repeated several times. Later, the animal is allowed to enter and explore either environment in the absence of the drug. A greater amount of time spent in the drug-associated environment appears to reflect positive reinforcing effects of the drug. In the aforementioned example, a greater time spent in the dark, rough-textured environment, compared with the light, smooth-textured environment, would suggest that the administered drug had positive reinforcing effects.

Whereas the acute positive reinforcing effects of drugs may be investigated using these paradigms, negative reinforcing effects can be examined by testing animals in the withdrawal or abstinent state. These paradigms also can be used to examine conditioned positive and negative reinforcement. For example, a rat can be trained to associate the presentation of alcohol with a light. The experimenter can then measure the frequency with which the rat presses a lever to turn on the light in the absence of alcohol (i.e., conditioned positive reinforcement).

### Neuroadaptation

Although the positive reinforcing effects of drugs are critical for *establishing* addictive behavior, both positive and negative reinforcing effects are probably important for *maintaining* drug use following the development of addiction. Neuroadaptive changes that occur with chronic drug use lead to increased positive and negative reinforcing effects. Thus, as mentioned previously, neuroadaptation is a modulatory process that can lead to increased reinforcement with repeated AOD exposure.

Sensitization is an increased response to a drug effect following repeated administration of the drug. Sensitization of drug effects that support further intake (such as the motivational state of “wanting” or “craving” and/or the physiological state of arousal) may contribute to the process of addiction. A recent conceptualization of the role of sensitization in drug dependence posits that a motivational state described as “wanting” is progressively increased by repeated exposure to drugs of abuse (Robinson and Berridge 1993). As “wanting” increases across repeated AOD exposures, the likelihood of relapse following periods of abstinence may increase, ultimately leading to compulsive drug use.

Counteradaptation refers to processes that are initiated to counter the acute effects of drugs. For example, tolerance is the reduction in a drug’s effect after repeated use of the drug. Tolerance to the desired effect of an AOD could stimulate increased AOD use in an attempt to re-experience the intensity of the drug’s initial effect.

Withdrawal is another counteradaptive process. In this case, the processes initiated to counter acute AOD effects are expressed when the drug is removed; thus the symptoms are often opposite in nature to the original drug effects.

## Neural Circuitry of Acute Drug Reinforcement

Information is passed between neurons by chemical transmitters, which are released and subsequently bound by receptive elements on neurons. This process leads to a cascade of intracellular events that changes the excitability of the cell and ultimately alters neuronal circuit activity. A circuit can be defined as a group of connected neurons that pass information related to a specific function. AOD’s hypothetically possess acute positive reinforcing effects because of their interactions with individual transmitter systems within the general reward circuitry of the brain. The intracellular events elicited by AOD’s can lead to changes in many other neural processes, including those that trigger the long-term AOD effects which eventually lead to tolerance, dependence, withdrawal, sensitization and, ultimately, addiction.

**Figure:**
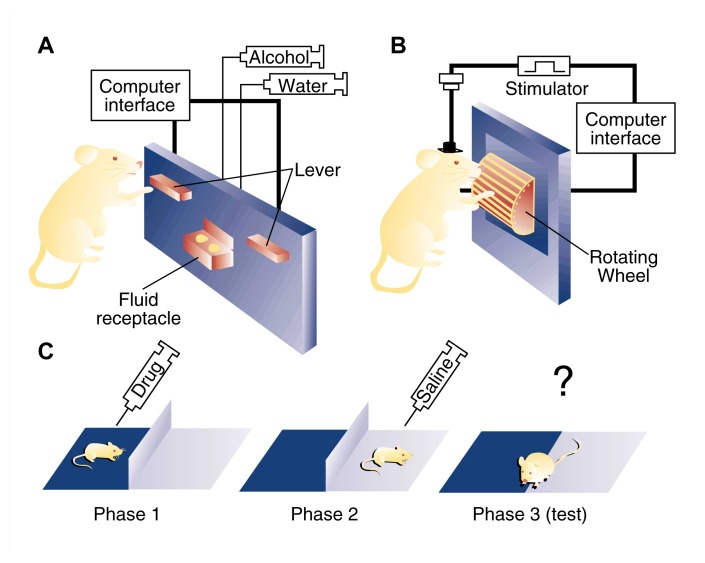
Animal behavioral paradigms used to explore the positive and negative reinforcing actions of alcohol and other drugs. (A) Oral alcohol self-administration paradigm, in which the animal is trained to press a lever to obtain alcohol instead of water. Rats will readily self-administer enough alcohol in daily 30-minute sessions to become mildly intoxicated. (B) Intracranial self-stimulation paradigm, in which the animal is trained to spin a wheel to receive a current through electrodes implanted in the brain. (C) Place-conditioning paradigm, in which injection of a drug is paired repeatedly with one environment and injection of a nondrug control solution (e.g., saline) is paired repeatedly with a different environment. The animal subsequently is allowed access to both environments in the drug-free state, and the amount of time spent in each environment is recorded. A greater amount of time spent in the drug-paired environment indicates a positively reinforcing drug effect.

The general reward circuitry of the brain centers around connections between the ventral tegmental area and the basal forebrain (which includes the nucleus accumbens, olfactory tubercle, frontal cortex, and amygdala). An important component of this system involves the transmitter dopamine; however, opioid, serotonin, and gamma-aminobutyric (GABA) systems are also involved. Evidence for a role of these systems in AOD addiction is discussed in the sections that follow. This discussion is not exhaustive, however: Other systems besides those discussed may play crucial roles in drug addiction processes. Moreover, individual systems interact with one another in complex ways that are beyond the scope of this overview.

### Dopamine Systems

The mesolimbic dopamine system connecting the ventral tegmental area and the basal forebrain appears to be critical to the self-administration of psychomotor stimulants (i.e., cocaine and amphetamine) (Koob 1992). The cell bodies of this dopamine system originate in the ventral tegmental area and send projections to the nucleus accumbens and basal forebrain, transmitting information to the dopamine receptors in these brain areas. This system also is important, but perhaps not critical, for reinforcement of opiate, nicotine, and alcohol use (Koob 1992; Pich et al. 1997). For example, alcohol consumption increases dopamine release in the nucleus accumbens from ventral tegmental neurons, and dopamine receptor antagonists^1^ reduce lever pressing for alcohol in rats. However, virtually complete destruction of dopamine nerve terminals in the nucleus accumbens failed to alter lever pressing for alcohol.

### Opioid Systems

Endogenous opioids are morphinelike neurotransmitters. Considerable evidence shows that the endogenous opioid systems have roles in the positive reinforcing effects of opiates (e.g., heroin). For example, Ettenberg and colleagues (1982) administered an opioid antagonist to rats previously trained to self-administer heroin. The animals reacted to this treatment by increasing their heroin intake, suggesting an attempt to compensate for the decreased efficiency of opioid neurotransmission.

The opioid system also appears to be important for the reinforcing effects of both alcohol and nicotine. For example, the opiate receptor antagonists naloxone and naltrexone reduce both alcohol and nicotine reinforcement in several animal models. Naltrexone has also shown success in decreasing alcohol consumption, frequency of relapse, and craving for alcohol in humans (O’Malley et al. 1992; Volpicelli et al. 1992). These data suggest that interactions between mesolimbic dopamine and opioid systems are important in the addictive process. (For review, see Koob and Nestler in press.)

### Serotonin Systems

The neurotransmitter serotonin helps regulate such functions as bodily rhythms, appetite, sexual behavior, and emotional states. Evidence indicates that serotonin systems are important in alcohol consumption. For example, increasing the level of serotonin in synapses or blocking certain serotonin receptor subtypes can decrease alcohol consumption (LeMarquand et al. 1994*a*, 1994*b*).

Serotonin systems also may be important in the acute reinforcing effects of drugs other than alcohol. For example, although reinforcement of cocaine use is attributed primarily to the dopamine system, cocaine strongly inhibits removal of serotonin from synapses, thereby facilitating increased binding of serotonin to its receptors (White and Wolf 1991). Antagonism of a specific serotonin receptor (i.e., 5-HT_2_) appears to decrease consumption of cocaine by rats (McMillen et al. 1993), and experimental activation of the serotonin 5-HT_1B_ receptor increases reinforcement by dopamine (Parsons et al. 1996).

### GABA Systems

Gamma-aminobutyric acid (GABA) is the primary inhibitory neurotransmitter in the brain. Sedative-hypnotic drugs including alcohol, benzodiazepines (e.g., Valium^®^), and barbiturates have long been hypothesized to modulate receptors in GABA systems. Supporting this concept, experimental drugs that decrease the function of GABA receptors reduce alcohol consumption by rats. Microinjections of GABA antagonists into various rat brain regions suggest that an important brain area for alcohol-GABA interactions is the central nucleus of the amygdala, a structure that communicates with the basal forebrain structures and is associated with emotion and stress. (For review, see Deitrich and Erwin 1996.)

## Mechanisms of Neuroadaptation

Neuroadaptations in the reward system accompany the development of addiction and can involve the same systems underlying acute drug reinforcement (within-system adaptation) or different systems (between-systems adaptation). These changes in the reward circuitry promote compulsive drug use in part by increasing a drug’s positive (e.g., sensitization) and negative (e.g., counteradaptation) reinforcing effects.

### Sensitization

Repeated administration of stimulants, opiates, or alcohol can result in sensitization, which appears to be mediated by the mesolimbic dopamine system (Wise and Leeb 1993). The process of sensitization, whereby an enhanced activation of dopamine function occurs in the mesolimbic system, may represent a within-systems mechanism of neuroadaptation. For example, injections of opiates or amphetamine directly into the ventral tegmental area that change the function of the dopamine neurons produce sensitization to later injections of these drugs in the periphery (White and Wolf 1991). As is the case with tolerance, sensitization may develop to one particular effect of a drug and not to another.

Another system that may have an important role in sensitization, representing a between-systems mechanism of neuroadaptation, involves corticotropin-releasing factor (CRF). This hormone is released by the hypothalamus and the amygdala in response to stress. CRF causes the release of additional stress hormones into the bloodstream from the pituitary gland (located at the base of the brain) and the adrenal cortex (located atop the kidneys). This stress-response system is called the hypothalamic-pituitary-adrenal (HPA) axis. The amygdala release may be responsible for behavioral responses to stress. Exposure to a variety of stressors can promote sensitization to drug effects, and the CRF-mediated stress-response system has been implicated in this sensitization. For example, stress hormones released by the adrenal cortex (i.e., corticosteroids) have been implicated in the increased locomotor response observed in mice following repeated administration of low doses of alcohol (Roberts et al. 1995).

Excitatory neurotransmitter systems also may represent a source of between-systems sensitization for AOD’s. The major excitatory neurotransmitter in the brain is glutamate. Administration of an antagonist of a specific glutamate receptor subtype can block the development of sensitization to psychomotor stimulants, suggesting a role for brain glutamate systems in sensitization (Wise 1988).

### Counteradaptation

Repeated AOD exposure also can lead to adaptations in the reward circuitry that oppose and neutralize a drug’s effects (i.e., counteradaptation). The persistence of these opposing effects after a drug has left the body may produce the motivational withdrawal response that possibly contributes to renewed drug use. As with sensitization, both within- and between-system adaptations appear to underlie counter-adaptation. Researchers have found decreased levels of dopamine in the nucleus accumbens during withdrawal from cocaine, opiates, and alcohol (Di Chiara and North 1992; Rossetti et al. 1992; Weiss et al. 1993); these results are opposite to those produced by acute exposure to these drugs. In addition, GABA transmission decreases and glutamate transmission increases during alcohol withdrawal, again reflecting the opposite effects of acute exposure (Koob et al. 1994).

As is the case with sensitization, the brain CRF systems and HPA axis may represent a between-systems source of counteradaptation. Rats exhibit a stress-like response when repeated administration of cocaine, opiates, or alcohol is terminated. In addition, alcohol-withdrawal–induced increases in anxietylike responses in rats were reversed by microinjection of a CRF antagonist into the central nucleus of the amygdala (Koob et al. 1994), and alcohol withdrawal is associated with increased levels of CRF in this brain region (Merlo-Pich et al. 1995). Altered corticosteroid activity also has been associated with both alcohol and benzodiazepine withdrawal. In mice, administration of corticosteroids exacerbated withdrawal convulsions, whereas a steroid synthesis inhibitor diminished them (Roberts and Keith 1995).

## Protracted Abstinence and Relapse

Perturbations in AOD reward pathways persisting after the acute withdrawal phase may promote vulnerability to relapse of drug-taking behavior. The scarcity of relevant animal models limits the study of the neurobiology of relapse. In one study, cocaine was withheld from animals trained to lever press for cocaine until the lever-pressing behavior was extinguished. The rats were then treated with drugs that activate the mesolimbic dopamine system and a rapid reinstatement of lever-pressing for cocaine was observed. (Stewart and deWit 1987).

Acamprosate, a medication that may modify glutamate action, is being marketed in Europe to prevent relapse in alcoholics. This drug has been shown to block the increase in drinking observed in rodents after forced abstinence (Spanagel et al. 1996; Heyser et al. in press). Similarly, opioid antagonists can prevent animals’ increased alcohol consumption caused by exposure to stress and have shown some success in preventing relapse in detoxified human alcoholics (O’Malley et al. 1992; Volpicelli et al. 1992). Finally, a recent study has found that agonists of a specific dopamine receptor subtype (i.e., the D_1_ receptor) can prevent “relapse” in abstinent rats previously trained to press a lever to obtain cocaine (Self et al. 1996). Although these studies suggest a role for dopamine, opioid, and glutamate systems in protracted abstinence and relapse, additional research using animal models is needed to provide a better understanding of the neurobiological mechanisms underlying the role of these systems in addiction.

## Extended Amygdala: Integrative Concept

Although the mesolimbic dopamine system is clearly important in drug addiction, its activity alone does not appear to account for the diversity of drug-reinforcement processes. Recent data suggest that the reinforcing actions of AOD’s may involve a neural circuit within the basal forebrain, termed the “extended amygdala” (Heimer and Alheid 1991). The extended amygdala comprises several basal forebrain structures—for example, the medial part of the nucleus accumbens and the centromedial amygdala. The extensive connections of this system to and from brain regions that are critical in various aspects of reinforcement support a role for the extended amygdala as the overall reward center of the brain.

The extended amygdala may regulate the acute reinforcing actions of AOD’s as well as neuroadaptations associated with addiction. Actions of the drugs of abuse on components of the extended amygdala are described above. Additional evidence includes the observation that acute administration of AOD’s produces increases in extracellular levels of dopamine in the medial nucleus accumbens (Pontieri et al. 1995). Also, neurons in the medial nucleus accumbens contain high levels of dopamine D_1_ and D_3_ receptor sub-types. Furthermore, the central nucleus of the amygdala appears to be important in acute alcohol reinforcement, as microinjection of GABA or opioid peptide antagonists into this brain region diminish lever pressing to obtain alcohol (Hyttia and Koob 1995).

Even more intriguing is the possibility of a role for the extended amygdala in counteradaptive processes associated with chronic drug exposure. A recent observation showed that microinjections of a GABA agonist into the central nucleus of the amygdala in alcohol-dependent rats decreased alcohol self-administration, whereas this treatment had no effect in non-alcohol–dependent animals (Roberts et al. 1996). These results suggest that the GABA system is altered significantly during the course of dependence. In addition, the interaction of CRF systems with alcohol withdrawal appears to involve the central nucleus of the amygdala (Koob et al. 1994). Thus, the extended amygdala may be involved in both acute AOD actions and various motivational aspects of addiction (Koob and Bloom 1988).

## Conclusion

The functional role of neurotransmitter systems and their integration into circuits contributing to addictive behavior are beginning to be elucidated. A focus is developing on a brain reward circuit that links the mesolimbic dopamine system and amygdala and on the neuroadaptive changes in neurotransmission that occur with chronic drug administration. Researchers also are investigating the genetic and environmental factors that may act on this circuit to influence individual differences in susceptibility to addiction. The resulting knowledge will enhance our understanding of the neurobiology of addiction and aid in the development of treatment therapies.

## Footnotes

1 For a definition of this and other technical terms used in this article, see central glossary, pp. 177–179.
